# Advancements, challenges, and prospects of explainable AI in sleep disordered breathing

**DOI:** 10.1007/s11325-026-03674-3

**Published:** 2026-04-30

**Authors:** Daniil Lisik, Tai Dinh, Ding Zou

**Affiliations:** 1 Department of Public Health and Clinical Medicine, Section of Sustainable Health, The OLIN Unit, Umeå University, Umeå, Sweden; 2 Krefting Research Centre, Institute of Medicine, Sahlgrenska Academy, University of Gothenburg, Gothenburg, Sweden; 3 Institute of Digital Technology, Thu Dau Mot University, Ho Chi Minh City, Vietnam; 4 Center for Sleep Research, Institute of Medicine, Sahlgrenska Academy, University of Gothenburg, Medicinaregatan 3, Box 400 , Gothenburg, 405 30 Sweden

**Keywords:** Deep learning, Machine learning, Obstructive sleep apnea, Precision medicine, Prompt, Treatable trait, XAI

## Abstract

Rapid advancements in artificial intelligence (AI), in combination with increased availability of rich large-scale clinical data, has paved the way for promising implementations in both diagnosing/subtyping as well as managing of sleep disordered breathing (SDB). A central strength of AI in this regard is how it facilitates analysis of complex multidimensional/modal data, for example pertaining to comorbidities and so-called treatable traits. However, the utility of such applications remains somewhat limited, as most AI models are so-called “black boxes”, for which it is not intuitive to assess how an output was arrived at. This poses challenges for ensuring patient trust and controlling bias. Explainable AI (XAI), which constitutes techniques to either distill “black box” models to simpler ones or to elucidate influential patterns by surrogate “white box” models, may provide such insights. Adoption of XAI within this field, however, is still at a nascent stage, and overall, the optimal role of AI alongside clinicians continues to be unclear. This review provides a clinically oriented overview of state-of-the-art implementations of AI and XAI in SDB. In addition, we discuss limitations and trade-offs with XAI and propose a general framework for personalized management of obstructive sleep apnea using XAI and treatable traits.

## Introduction

Artificial intelligence (AI) is rapidly increasing in competence, particularly so deep machine learning (ML) algorithms, which enable (semi-)autonomous extraction of complex non-linear patterns from vast data. In sleep medicine, some of the first implementations were ML algorithms for automatic sleep staging, using e.g., neural networks, which carry the benefit of being able to process raw signal as input [1]. Despite promising advancements in certain areas of medicine, and the evident versatility of such applications, the clinical utility of AI within the healthcare sector remains limited [2]. In sleep medicine, implementation of AI has been assessed for screening/diagnosis, clinical decision-making, and patient communication [3, 4]. For example, fine-tuned large language models (LLMs) have been shown to outperform clinicians in generating differential diagnoses for challenging clinical cases [5], and various ML algorithms have been successfully trained to diagnose/classify specific diseases [6–8]. Novel meaningful subtypes may be discovered using ML by enabling automation of various processes, such as sleep study scoring and analysis of complex multi-omics data [9]. Similarly, ML can further the efforts to provide personalized and data-driven management, exemplified by high prediction accuracy in modern algorithms [3]. These technologies may also be used for “soft” tasks, such as patient education by distilling complex medical information into common language [10–12]. Such results have also been reported within the field of sleep-disordered breathing (SDB), including for diagnosis [13] and prognosis [14] using deep learning algorithms. The literature is relatively scanty, however, in comparison to many other fields, and mostly consists of studies focusing on obstructive sleep apnea (OSA) [15].

Despite promising findings, several unmet challenges remain before AI can be broadly and deeply incorporated into clinical practice within sleep medicine. One of the major challenges is that most ML algorithms, particularly those incorporating deep neural networks, are so-called “black boxes”, for which the “reasoning” (i.e., how the output/prediction was arrived at) is non-intuitive/complex to uncover [16]. This gives rise to challenges with controlling bias, ensuring appropriateness of output, and defining the scope of responsibility of AI [4]. Numerous explainable AI (XAI) techniques have been developed in response, but their utility and optimal role along clinicians is still unclear. This review aims to provide a practical hands-on synthesis of the latest developments of XAI in SDB. An extensive literature review in Google Scholar, PubMed, and ScienceDirect using comprehensive search queries, including spelling variation/synonyms and controlled vocabulary terms, focusing primarily on papers published in the past 5 years, constituted the basis for this review. We focus on cutting-edge implementations and techniques, as well as issues and potential remedies for balancing performance and explainability, equity, and privacy, ultimately aiming to answer the following questions: (1) how can AI be used in the field of SDB *today*? (2) how can we elevate AI-based/assisted management of SDB patients *in the future*?

## Primer on AI and XAI

AI is a broad term, technically encompassing any software with the ability to perform tasks or solve problems that are attributable to requiring intelligence [17]. ML constitutes a subset of AI, distinguished by the ability to learn from data without explicit programming/instructions [18]. ML is particularly useful in clinical contexts, as the associations and patterns therein are often intricately tangled and non-linear, easily extending beyond what is practical for humans to overview and put into explicit codes and systems of rules.

ML approaches can be divided by the learning mechanism. In *supervised learning*, “ground truth”/output labels are fed to the model, together with characterization data from which the model learns how to assign appropriate output values (categorical: classification, continuous: regression). In *unsupervised learning*, on the other hand, the model does not have access to any labels, rather, it learns the inherent patterns of the data, with the aim to reduce dimensionality of the data or, more commonly, segment the data into distinct subgroups (cluster/trajectory analysis). Between these two forms of learning is *semi-supervised* learning, where a portion of the data are labelled to assist the model in the initial learning (which subsequently applies to the unlabeled data). Model feedback may also be provided iteratively, as is done in *reinforcement learning*, where either a human or an artificial environment provides feedback to the model [18].

**Fig. 1:**
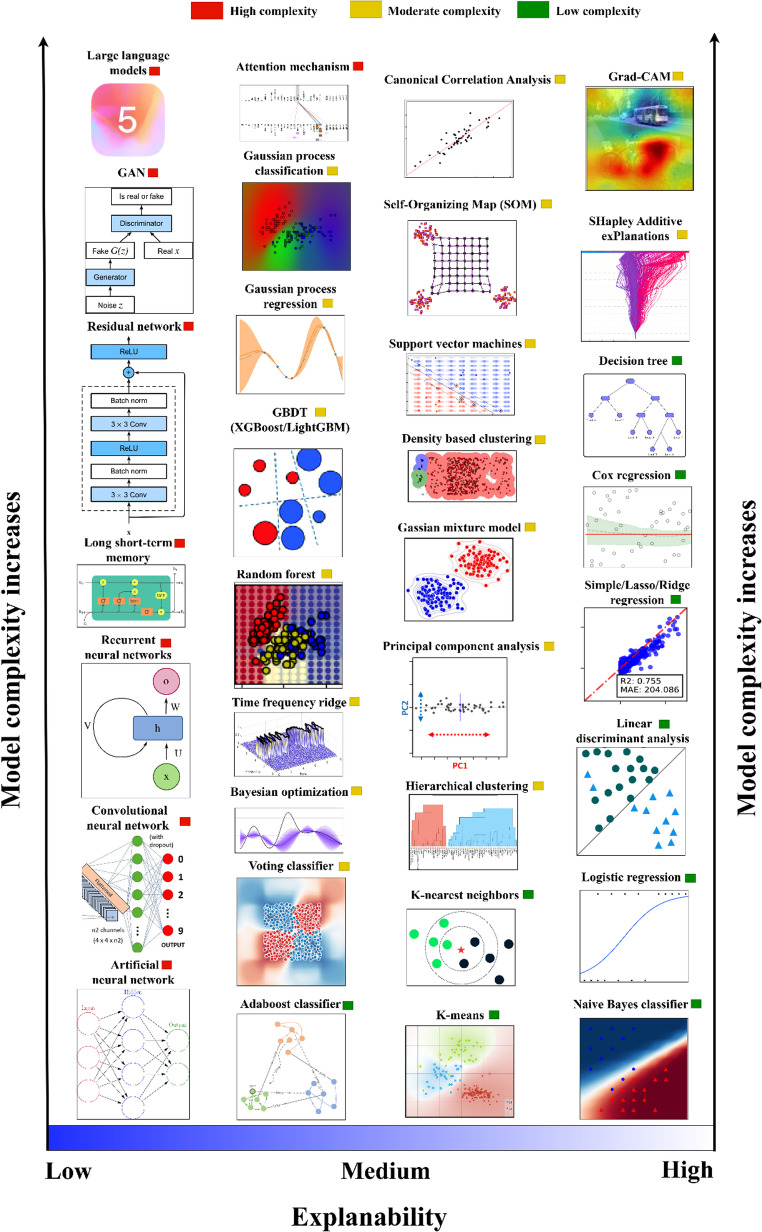
Common machine learning algorithms ordered by explainability (increasing from left to right on the x-axis) and model complexity (increasing bottom to top on the y-axis)

Another way of stratifying ML approaches is by the architecture of the model. A stratification with relevance for clinical implementation is “white-box” (also called transparent models) and “black-box” models. The “rationale” (i.e., algorithmic pathway from input to output) of white-box models can be understood inherently (by design) and usually quite intuitively so. White-box models are largely constituted by more or less “simple” algorithms, such as logistic regression and decision trees [19]. Black-box models, on the other hand, cannot be intuitively interpreted by design, due to their substantially more complicated algorithmic architecture [20]. This renders interpretability low, but at the same time allows for learning of highly complex patterns [21], particularly with large amounts of training data [18]. Complexity and explainability of common ML models is illustrated in Fig. 1.

XAI is a form of AI which aims to provide high-level understanding of how the output of an ML model is attained. XAI can be divided by whether the explanation technique is embedded withing the actual ML algorithm (“intrinsic”, i.e., white-box model) or if it utilizes techniques to explain an otherwise black-box model, often after running the model (“post-hoc”). The latter type of algorithm may also be divided into techniques that allow for “explanation” locally (e.g., individual subjects) or globally, i.e., of the entire model, as well as by whether the technique is applicable to any ML algorithm (“agnostic”) or if they are only applicable to specific types of ML algorithms (“model-specific”) [19]. The development of XAI has seen a rapid increase in recent years [22], but implementation within clinical research (and even more so clinical practice) remain at a nascent stage, and the efforts of establishing these techniques is further hampered by the different definitions of “explainability” [23] and lack of standardized methods for assessing performance of XAI techniques [24]. The term itself (“explainability”) lacks a formal and widely accepted definition [23]. In this work, we refer to “explainability” as the understanding of *why* a model produces an output given specific input (high-level understanding of model), and “interpretability” as the understanding of *how* a model obtains an output at a low(er) level, based on its algorithmic architecture [24].

## AI and XAI in SDB

### Screening, diagnosis, and subtyping/classification

A prerequisite for optimal management is that individuals needing treatment are indeed identified, which constitutes a challenge in SDB. For example, OSA remains underdiagnosed despite awareness of its serious implications [25]. Several factors likely explain this, one of which being that the clinical presentation of OSA can vary between individuals and different groups, such as women, in which atypical and nonspecific symptoms often result in delay or even lack of diagnosis [26]. OSA severity may also exhibit considerable night-to-night variability, making single-night assessments prone to misdiagnosis/misclassification [27]. Furthermore, the different underlying pathophysiological pathways and intricate associations with comorbidities give rise to numerous phenotypes [28], which are burdensome to distinguish through traditional methods of assessment and manual classification. There is also an important practical challenge in that OSA is highly prevalent, affecting around 1 billion adults worldwide [29]. Central sleep apnea (CSA), while substantially less common in the general population, is prevalent in certain conditions, such as heart failure, following stroke, and among users of opioids [30]. Although the gold standard method for diagnosis of these conditions (as well as differentiation from other sleep disorders) is in-lab polysomnography (PSG, type I sleep study) [31, 32], home sleep apnea tests (HSAT, type II-IV sleep study) are largely reliable alternatives [33], suitable not least in contexts where in-laboratory PSG is not feasible, e.g., due to cost or practicality. Similar challenges are present for classification/subtyping of SDB, as this typically also necessitates resource-intensive examination. Here, AI is of high relevance, significantly expanding our ability to distinguish likely cases from non-cases as well as better characterize patients through the ability of such algorithms to analyze vast amounts of complex (multi-modal) data and identify latent patterns therein.

Multiple promising implementations have been reported for screening purposes. Utilizing AI-based pulse oximetry-monitoring with type IV devices, it is possible to achieve accuracy in diagnosing moderate/severe OSA comparable to that of PSG (accuracy of > 90%) [6, 34]. Similar performance has been seen with AI-based analysis of audio recordings [35], as well as AI-based analysis of multi-modal input, e.g., pulse oximetry combined with demographic, anthropometric, and clinical data [36]. High accuracy has also been noted for AI-based applications for OSA severity assessment based on anthropometric and questionnaire data [37].

Plenty of data-driven exercises have also been performed to elucidate phenotypes [28], expanding our understanding of the complex heterogeneous disease patterns of OSA. Comorbidities, for example, exhibit diverse patterns across OSA severity strata (as per apnea-hypopnea index [AHI]) [38]. Adverse outcomes have also been linked to AI-derived phenotypes such as OSA with excessive daytime sleepiness, prompting increased awareness and unearthing potential for targeted treatment [39]. However, many classification exercises merely produce “phenotypic makeup” flavors and lack clear clinical utility, e.g., by not evaluating the association between derived subgroups and clinical outcomes and actionable risk factors [40]. Also, as has been demonstrated in the existing literature that the very selection of input variables is highly influential on the derived subgroups [38]. Most cluster analyses are largely hypothesis-driven in terms of selected input variables, which diminishes the data-driven aspect of AI-based phenotyping and ultimately limits discovery of truly novel subgroups.

Endotyping (identifying likely disease mechanisms) of OSA is of substantial clinical relevance, given how such information facilitates personalized treatment. This is of particular importance in OSA as the gold standard treatment of continuous positive airway pressure (CPAP), while effective, is hampered by low adherence [41]. Typically, such assessment is done in detail only in rare cases, as it requires invasive and time-consuming examinations. Although AI applications for this aim are still in their infancy, with mixed performance (for example, a decision tree model predicted loop gain and muscle responsiveness with accuracy at chance level) [42], promising data have also been published, indicating that with continued improvement in model architecture and larger training data, AI algorithms analyzing routine PSG data may soon reliably detect various pathophysiological mechanisms [43], and do so at scale [44]. In summary, we see that AI is already a powerful tool to aid physicians in identifying and subtyping patients, but these technologies may assist throughout the full patient management journey, as will be discussed in the following section.

### Management and prognosis

Following an appropriate diagnosis, it is essential to select a suitable management strategy and continuously re-evaluate treatment adherence, efficacy, as well as potential changes in relevant comorbid conditions, lifestyle, and risk factors. Here, again, AI is a powerful tool to aid physicians in their decision-making. For example, oral appliance therapy (OAT), which is a common second-line treatment alternative in OSA, is less effective in maintaining airway patency compared to CPAP. Careful selection of suitable patients for this treatment modality is thus needed. In a study of a supervised learning algorithm, using PSG, age, and body mass index (BMI) as input, a mean accuracy of 91% in predicting OAT-responders (AHI < 5) was achieved [45]. Unsupervised learning can also be of use in this context, as it has been reported that certain AI-derived OSA phenotypes exhibited substantially lower rates of successful CPAP treatment [46]. Such insight could increase the inclusion of patients’ preferences and expectations of treatment in a data-driven and efficient fashion, and further optimize initial impression and treatment setup, which is of high importance for adherence [47].

Beyond subjective treatment response and satisfaction, CSA and OSA increase the risk of various adverse long-term outcomes, including mortality [31, 48]. At the same time, traditional statistical endeavors of e.g., the role of CPAP in protecting against such outcomes have resulted in mixed findings [49]. Incorporating richer (longitudinal) data into sophisticated AI algorithms may likely facilitate a more comprehensive risk assessment for individual patients. Several algorithms have shown promising accuracy for long-term (all-cause) mortality prediction, using relatively easily accessible data [50], particularly so in distinguishing high- and low-risk subpopulations [51].

**Fig. 2:**
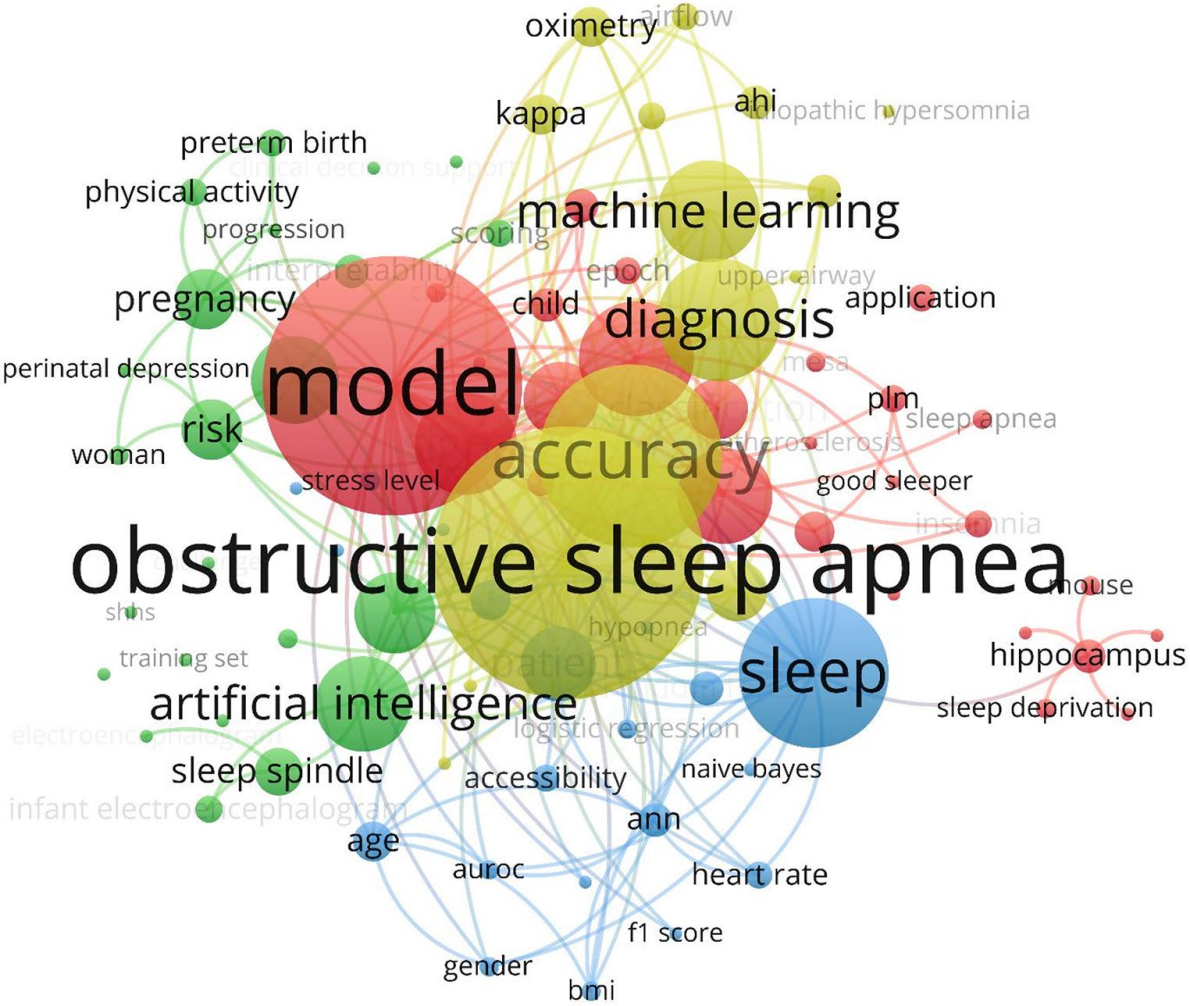
Keyword co-occurrence map (titles/abstracts). Node size indicates frequency; edge thickness indicates co-occurrence strength. Four clusters emerge: yellow—core SDB diagnostics (obstructive sleep apnea, polysomnography, oximetry, sleep staging); blue—methods/cohorts (random forest/ANN/naïve Bayes, evaluation metrics, heart rate, age/BMI/gender); green—clinical translation & explainability (AI, interpretability, clinical decision support, risk, biomarkers, EEG features); red—signal-based detection & related mechanisms (model, ECG/signal, classification; insomnia/neuroscience terms)

Figure 2 presents a four-cluster VOSviewer map of the XAI-in-SDB literature. The yellow cluster centers on core diagnostics—*obstructive sleep apnea* linked with *polysomnography*,* oximetry*,* airflow*,* sleep stage*,* diagnosis*, and *accuracy/performance*. The blue cluster captures methodological and cohort terms, grouping learning algorithms (*random forest*,* ANN*,* naïve Bayes*,* logistic regression*), evaluation metrics (*AUC*,* F1*), physiologic features (*heart rate*), and demographics (*age*,* BMI*,* gender*). The green cluster reflects clinical translation and explainability, connecting *artificial intelligence*,* interpretability*,* clinical decision support*,* risk*,* biomarkers*, and population contexts (e.g., *pregnancy*,* child*, EEG features like *sleep spindles*). The red cluster emphasizes signal-based detection and related pathophysiology, spanning *model*,* electrocardiogram*,* signal*,* classification*,* respiration/insomnia*, and a neuroscience subtheme (*hippocampus*,* prefrontal cortex*,* mouse*).

Representative studies on AI and XAI applications in sleep-disordered breathing, including study objectives, explainability methods, performance, and key findings, are summarized in Table 1.

**Table 1:** Machine learning techniques utilized in studies on SDB

Reference	Cohort/study population	Study objective and input data	Explainability technique(s) used	Performance	Key findings
Screening, diagnosis, and classification of SDB					
Álvarez et al. 2020 [52]	Unattended PSG recordings from 239 patients with suspected OSA.	Regression on oximetry + airflow signals estimates AHI.	Moderate explainability with SVM.	Estimated vs. actual AHI showed intra-class correlation = 0.93, outperforming single-channel methods.	ML on combined oximetry + airflow markedly boosts OSA screening accuracy—especially in severe cases—over single‑channel methods.
Shi et al. 2023 [53]	1,656 subjects who underwent PSG at the Second Affiliated Hospital of Xi’an Jiaotong University, China, between 2018 and 2021.	Severe OSA classification/prediction.	High explainability. LR, GBM, and SHAP offer clear explanations for model predictions.	GBM led with AUC 0.857, accuracy 0.766, sensitivity 0.798, specificity 0.734.	Interpretable GBM predicts severe OSA from clinical + questionnaire data; SHAP flags waist/neck size, Epworth sleepiness scale score, age, and Berlin questionnaire score as top predictors.
Bahr-Hamm et al. 2023 [54]	Data from full-night PSG recordings of 86 patients.	OSA severity classification.	Moderate explainability with SVM.	Snoring‑signal entropy led with AUC ≤ 0.84 for non‑ vs. severe OSA, while ECG‑VLF and thoraco‑abdominal effort entropy also performed well.	Entropy of snoring, ECG‑VLF, and thoraco‑abdominal effort signals can screen and grade OSA as PSG surrogates, with snoring entropy performing best.
Han et al. 2023 [55]	4,014 patients who visited the sleep clinic of Samsung Medical Center between 2014 and 2021.	OSA severity classification/prediction.	Moderate explainability: traditional ML (RF, gradient boosting) and unsupervised methods (HC, KM) offer varying transparency.	Models achieved 88% accuracy at AHI ≥ 5, 88% at AHI ≥ 15, and 91% at AHI ≥ 30.	Combining supervised + unsupervised ML predicts OSAS severity better than standard approaches, enabling efficient screening without extensive tests.
Dai et al. 2024 [56]	610 patients underwent PSG at the Sleep Medicine Center of the Second Affiliated Hospital of Fujian Medical University between Jan 2021 and Dec 2022.	Moderate-to-severe OSA classification/prediction.	Moderate explainability: combines LR, NB, SVM, RF, DT, and ANN.	ANN led performance with AUROC 0.804, accuracy 69.9%, recall 86.5%, specificity 61.5%, precision 53.2%, and F1 65.9%.	Six ML models using age, gender, BMI, and mean sleep heart rate predict moderate‑to‑severe OSA; the ANN model performed best and is primed for a cloud‑based mobile sleep platform.
Massie et al. 2025 [57]	266 suspected OSA subjects underwent simultaneous PSG and PAT‑based HSAT recordings.	OSA severity classification.	Highly explainable: a decision‑tree model gives interpretable scores for each respiratory event.	Panorama topped context‑unaware autoscoring in OSA severity accuracy by 9% (3% rule) and 7% (4% rule).	Context‑aware Panorama boosts P‑HSAT accuracy and supplies event‑level rationales, streamlining reliable home OSA diagnosis.
Bulut Eris et al. 2025 [58]	Data on 466 subjects: 234 OSAS patients and 232 controls.	OSAS‑control classification.	Moderately explainable ML models: DT, SVM, KNN, ensemble DT, and neural network.	Neural network using five flow‑volume features reached 97.1% accuracy.	With five flow‑volume PFT features, this study built a high‑accuracy OSAS detector that’s quicker and cheaper than PSG.
Chao et al. 2025 [59]	29 Taiwanese patients scheduled for SDB surgery.	OSAS severity classification (mild, moderate, severe).	Moderate explainability: blends traditional/automatic linear regression, CR, and signal processing.	CR model classified OSAS severity with 90% accuracy.	Neck‑worn piezo sensor merging snore and carotid pulse signals improves OSAS diagnosis.
Li et al. 2025 [60]	60 male OSA patients and 60 male controls.	Classify OSA patients vs. controls and predict symptom severity.	Moderate explainability: classification uses SVM, logistic & linear discriminant analyses, RF, gradient boosting, and DT.	Merging static + dynamic connectivity sharpened classification—SVM excelled—and explained 30% of cognitive impairment, 56% of sleepiness, and 28% of anxiety/depression.	Static + dynamic cerebrocerebellar connectivity accurately classifies and predicts OSA, pointing to compensatory reorganization and disrupted cognitive networks as neuroimaging markers.
Fang et al. 2025 [61]	465 participants, 1,495 simulated‑snore recordings.	OSA severity classification from simulated snoring.	Moderate explainability: combines SVM, KNN, RF, and AST.	AST deep model topped classic ML with accuracies of 0.926, 0.887, and 0.830 at AHI cutoffs 5, 15, and 30 events/hour.	DL models accurately detected OSA from simulated snores, outperforming traditional ML.
Balci et al. 2022 [62]	PSG data (Pressure Flow, ECG, Pressure Snore, SpO2, Pulse, Thorax) from 19 patients.	SDB type classification	Moderate explainability: ANN, SVM, RF, NB, KNN, DT, and LR offer differing levels of transparency.	Random forest scored 76.3% on the 5‑class task; excluding Hypopnea boosted accuracy to 86.6% for 3 classes.	System classifies four SDB types from six PSG signals; time‑ and time‑frequency features prove effective.
Parbat et al. 2024 [63]	Apnea-ECG dataset from Physionet.org	Classification of sleep apnea events	DT, RF, SVM, AdaBoosted DT, voting, and stacked models are moderately interpretable, but multiscale-entropy features add complexity and limit clarity.	Accuracy: 74.3%, Sensitivity: 53.8%, Specificity: 82.7%, AUC: 69.0%	Sleep apnea detected via multiscale-entropy on single-lead ECG/respiration, yielding balanced, moderate accuracy ideal for wearables.
**Automated PSG annotation and sleep staging**					
Palotti et al. 2019 [64]	Multi-Ethnic Study of Atherosclerosis (MESA) Sleep dataset	Classification of sleep-wake stages	Moderate explainability. Traditional algorithms and simpler ML models offer some explainability, while deep learning models (CNN, LSTM) are less transparent.	Top DL models (LSTM 100, CNN 100) reached ~ 88% accuracy, beating traditional methods and the device’s built‑in algorithm.	Using the large MESA Sleep dataset, this benchmark shows modern ML/DL algorithms far surpass traditional methods for sleep‑wake classification and sets a foundation for future research.
Nasiri 2020 [65]	Sleep Heart Health Study and Physionet 2018 Challenge (P18C).	Classification of sleep stages	Adversarial, attention‑based CNNs add some interpretability, but overall explainability stays moderate.	On P18C, the method lifted precision 0.72 → 0.84, sensitivity 0.74 → 0.85, and κ 0.64 → 0.80.	A multi‑adversarial attention network yields transferable EEG features and decisively tops prior models in cross‑dataset sleep staging.
Kang et al. 2021 [66]	40 subjects gathered from full in-lab PSG recordings.	Classification of sleep stages	MLP captures complex patterns but offers only moderate interpretability.	Cohen’s Kappa rose by 0.28 over automated scoring, while scoring time dropped 60% versus full manual review.	Adding uncertainty estimates to automated sleep staging boosts accuracy and efficiency, letting clinicians focus on doubtful epochs.
Dutt et al. 2023 [67]	Sleep-EDF (Sleep-EDFx, 2013 version).	Multi-class sleep stage classification	CNN‑CRF offers moderate interpretability; Grad‑CAM visualizes key EEG features for explainability.	86.81% accuracy on Fpz‑Cz EEG, with N1 F1‑score rising to 63.05% over baselines.	SleepXAI—a CNN‑CRF model with modified Grad‑CAM—achieves state‑of‑the‑art accuracy (especially for N1) and provides visual explanations of its decisions.
Ingle et al. 2024 [68]	MESA (Multi-Ethnic Study of Atherosclerosis) dataset.	Classification of sleep stages	High explainability: SHAP offers clear insights into each feature’s role in sleep stage classification.	Overall accuracy was 83.8% on the full MESA dataset, ranging from 84.5% to 87.3% across subgroups (insomnia, PLM, apnea, healthy).	Wavelet-based, XAI-enabled sleep scoring accurately classifies stages—even in disordered groups—outperforms state-of-the-art models and reveals key features.
McCausland et al. 2025 [69]	PhysioNet 2018 “You Snooze, You Win” and WSC datasets.	Sleep‑stage transition classification.	Time‑frequency ridge + SVM yield moderately interpretable sleep‑stage features.	Proof‑of‑concept model: 92.5% accuracy, 93.8% sensitivity, 91.2% specificity.	Consistent time‑frequency patterns at stage shifts could enhance inter‑scorer reliability and automated scoring.
**Endophenotyping and risk stratification**					
Tondo et al. 2024 [70]	Data collected from patients referred to the authors’ unit since 2005, forming a local healthcare registry.	Clustering OSA patients into phenotypes and predicting mortality risk based on phenotype characteristics.	OSA patients were clustered into distinct phenotypes whose characteristic profiles predict mortality risk.	Silhouette-based clustering yielded three OSA phenotypes. Mortality prediction: NN—AUC 0.948, accuracy 0.818, F1 0.818; LR—AUC 0.947, accuracy 0.828, F1 0.828.	Three distinct OSA phenotypes emerged, and nocturnal hypoxemia independently predicted mortality beyond AHI.
Ai et al. 2024 [71]	Sleep Heart Health Study, Osteoporotic Fractures in Men Study Sleep study.	Predict long-term CVD risk and mortality based on disrupted delta wave activity during sleep	Moderate explainability: Cox and XGB offer moderate interpretability, with SHAP providing local explanation for predictions.	Lower delta wave entropy predicts higher coronary heart disease, CVD, and mortality risk, and boosts model performance beyond standard sleep metrics.	Low delta wave entropy—a sign of disrupted delta activity—better predicts CVD risk and mortality than traditional sleep measures.
**Clinical management**,** patient-reported outcome measures (PROMs)**,** and treatment response**					
Kim et al. 2021 [72]	163 OSA subjects who underwent sleep surgery at a single institution.	Predicting OSA surgical outcomes.	Moderate explainability with random forests and gradient boosting.	Gradient boosting topped accuracy at 0.708, beating logistic regression (0.542) and physicians (0.522).	Gradient‑boosting ML predicts OSA surgery outcomes from demographic, anatomical, and sleep data, guiding treatment and avoiding unnecessary operations.
Kaplan et al. 2017 [73]	Sleep Heart Health Study; middle-aged to older adults	PSG plus demographic + clinical data used to predict subjective sleep quality.	LASSO: high RF: moderate	Models explained little variance in subjective sleep quality (R² ≈ 7–13% with lasso; 8–9% with random forest).	Standard PSG and qEEG give limited insight into subjective sleep quality.
Ravindra et al. 2023 [74]	1,083 pregnant individuals with 2,305 measurements of physical activity and sleep data.	Predicting gestational age from one-week wearable actigraphy (activity and sleep) data.	Moderate explainability: combines ResNet-based deep learning and traditional ML, emphasizing interpretability via feature attribution and error analysis.	Series2Signal outperformed other ML methods, achieving a 7.52‑week MAE in gestational age prediction.	Wearable deviations in activity and sleep correlate with preterm birth risk, and gestational age prediction errors can flag high‑risk pregnancies.
Garbazza et al. 2024 [7]	Data from the Life-ON project, a multicenter cohort study on sleep and mood changes during the perinatal period	Classification predicts perinatal depression risk.	Moderate explainability with SVM.	SVM (cross‑validated): AUROC 0.774, AUPRC 0.388, sensitivity 0.528, specificity 0.826.	ML model predicts perinatal depression risk from early‑pregnancy data, with insomnia, poor sleep quality, daytime sleepiness, and psychiatric/psychosocial factors as top predictors.
Wahab et al. 2024 [75]	SaYoPillow	Stress‑level classification.	Moderate explainability: trees & SVM are clear, but MLP, XGB, and LLM cut transparency.	SVM scored 99.37% accuracy, surpassing the decision tree (98.41%), MLP (97.62%), XGBoost (98.73%), and earlier models on the same data.	SereniSens merges physiological signals, ML, and a tuned GPT‑3.5 Turbo to predict sleep‑time stress, outperforming prior methods on the same data.

LASSO: Least Absolute Shrinkage and Selection Operator, RF: Random Forest, SVM: Support Vector Machine, LR: Logistic Regression, DT: Decision Tree, Grad-CAM: Gradient-weighted Class Activation Mapping, KNN: k-Nearest Neighbors, MLP: Multilayer Perceptron, XGB: Extreme Gradient Boosting, CR: Categorical Regression, AST: Audio Spectrogram Transformer, PCA: Principal Component Analysis, NB: Naïve Bayes, SHAP: SHapley Additive exPlanations, CRF: Conditional Random Field, HC: Hierarchical Clustering, CNN: Convolutional Neural Network, LSTM: Long Short-Term Memory network, ANN: Artificial Neural Network, GBM: Gradient Boosting Machine, KM: k-means Clustering, Cox: Cox Proportional Hazards Regression, BO: Bayesian Optimization, OSAS: Obstructive Sleep Apnea Syndrome

## Why XAI? And how explainable should it be?

Although the body of evidence demonstrating the many benefits of incorporating AI in various contexts of SDB is ever-increasing, several unmet challenges remain, some of which concern the very foundation of many commonly used ML algorithms (Table 2). Black-box models impede understanding of how the output/prediction is arrived at. This in turn can mask potential biases. As an example from another field, an ML model training on identifying malignant skin lesions turned out to be influenced by the presence of rulers in the image (an artefact stemming from the measurement of tumors in the training material and not an actual feature of the skin) [76]. Such issues can very well extend or present as erroneous classification or management recommendations, and to prevent/counteract this, it is essential to understand ML models, so that biases and errors may be identified and the model be fine-tuned as appropriate (e.g., to counteract biases or to update the training data when new relevant information is made available). With increasing incorporation of AI in clinical practice, understanding how these models “reason” will be essential to instill confidence in patients and physicians and enable the former to provide informed consent and the latter to retain their professional accountability. This is especially relevant as LLMs (which are already widely evaluated to act as co-assistants in various clinical contexts) [77] may – either by design or by malicious prompt injections/malware – hide, lie, and deceive, a phenomenon referred to as scheming, which has already been noted with most major frontier LLMs [78]. There is also a juridical dimension to this, as e.g., the European General Data Protection Regulation (GDPR) states that individuals have a right to be informed of how their data are used in automated decision-making processes, including underlying mechanisms in use and significance/consequences [24]. From a modelling/performance perspective, it is also important to note that while many of the most impressive discoveries and developments within (applied) AI in recent years has been through black-box models, they are not necessarily better suited for every task or context. While deep neural networks (DNNs) may extend their capability of learning complex patterns with increasingly large training data beyond that which is feasible with traditional white-box models [18], it has been argued that in clinical contexts, particularly with structured data containing meaningfully selected variables, there is not necessarily a trade-off between accuracy and transparency of the model “reasoning” [4].

**Table 2:** Technical, clinical, and ethical challenges, bias, and performance in XAI vs. black boxes

Bias, challenge, or performance aspect	Definition	Black-box	Case study of XAI in OSA (or related subfield in sleep medicine, if available)
Sample bias	Training data not representing the target population, particularly damaging in case of clinically relevant underrepresented groups	Challenging to uncover at design/development phase (e.g., through external validation), although indicators may be present in e.g., descriptive statistics	Relatively easily identifiable and amendable, e.g., through feature importance techniques [79].
Representation bias	Features/labels don’t faithfully capture the clinical construct across groups (measurement/encoding/preprocessing mismatch).	Usually hidden until subgroup audits; aggregate metrics may look fine; reliance on site/device/proxy artifacts is opaque.	More readily flagged via attributions/saliency/counterfactuals/TCAV showing proxies/artifacts or label inconsistencies; mitigation still needs data/feature standardization, not explanations alone. [80].
Reliability and generalizability	Reliability: stable, reproducible outputs under small perturbations/retraining and overtime. Generalizability: consistent performance across sites, devices, populations, and prevalence shifts	Brittleness and shortcut learning are opaque; domain shift and threshold drift are hard to anticipate or diagnose without extensive external validation.	Explanations can flag instability and shift (e.g., changes in feature attributions across runs/sites, counterfactual/perturbation tests) and expose non-causal dependencies; however, explanations must be checked for fidelity and stability. Mitigation still needs diverse data, calibration/uncertainty monitoring, and periodic revalidation [81].
Historical bias	Influence of past discriminatory practices and beliefs	Challenging to uncover at design/development phase (e.g., through external validation), although indicators may be present in e.g., descriptive statistics	Relatively easily identifiable and amendable, e.g., through Local Interpretable Model-Agnostic Explanations (LIME) [82].
Equality and fairness	Equitable performance and treatment across demographic/clinical groups; absence of unjustified disparate impact.	Proxy use and subgroup harms are hidden; issues surface late via external audits; threshold trade-offs and fairness metrics are hard to justify.	Explanations reveal proxy reliance and group-specific reasoning (subgroup attributions/SHAP, TCAV, counterfactuals), aiding audits (e.g., equalized odds, calibration) and threshold setting; mitigation still requires data/process changes (reweight/relabel, fairness constraints, group-wise calibration) and governance [82, 83].
Measurement bias	Systematic error or misrepresentation of input data, e.g., due to actual measurement or preprocessing (e.g., labelling, scaling)	Data are often inputted as-is, in which case identifying the culprit may be challenging. If proper explorative data analysis is performed, identification and amendment may be feasible.	Relatively easily identifiable and amendable, e.g., through feature importance techniques [79].
Confirmation bias	Does the model merely aim to confirm a predetermined hypothesis, through its implementation or input data selection/preprocessing?	Through its opaque design, there is less risk of (re)affirmative finetuning/selection of final model based on predetermined hypotheses. Ultimately, however, selection of variables and preprocessing holds the strongest weight here.	By design it is possible to unearth and correct such bias, but at the same time, XAI is also more likely subjective to finetuning/selection of final model based on predetermined hypotheses. Ultimately, however, selection of variables and preprocessing holds the strongest weight here [84].
Algorithm bias	Systematic error due to algorithm architecture or implementation thereof	Depending on underlying cause (e.g., sampling bias) evaluation and appropriate configuration may be feasible.	Depending on underlying cause (e.g., sampling bias) evaluation and appropriate configuration may be feasible. Due to the nature of XAI applications, debugging this matter is likely easier.
Clinical efficiency and effectiveness	How well does the model perform? Is it accurate and does it provide efficiency to management of patients?	With relatively few exceptions (mostly due to insufficient training data size or improper implementation) superior to XAI in terms of pure clinical performance, although long-term efficiency may be comparable or even inferior in complex clinical cases.	Explanations can speed up validation, surface failure modes for quick fixes, support clinician–patient communication, and enable targeted quality assurance—potentially improving time-to-report and treatment. On the other hand, performance limitations may partly outweigh benefits [83].
Patient safety	How well can the model guarantee patient safety and be held “responsible” (e.g., through transparent trackback, explanation of reasoning, etc.)?	Limited possibilities to understand reasoning of model, although empirical evaluation of safety may be performed (typically costly and time-consuming).	Although no model (nor clinician for that matter) can provide complete guarantee of success or patient safety, XAI can provide insight into model reasoning and patient safety through various intrinsic and post-hoc explanation techniques.
Data augmentation bias	Bias introduced by synthetic/transformations that distort distributions or encode assumptions (e.g., oversampling, time-warping).	Model may overfit to augmentation artifacts; impact appears only under distribution shift.	Explanations/counterfactual tests reveal reliance on augmented artifacts; mitigation: balanced, label-preserving augmentations with ablations and invariance checks [79].
Generative bias	Bias from generative components/outputs (e.g., synthetic data, auto-reports) driven by training data, prompts, and decoding.	Plausible outputs hide bias/hallucinations; causes hard to trace without intensive audits.	Can probe/flag via log-prob analysis, concept probes (e.g., TCAV), counterfactual prompts, and attribution; mitigation needs curated data/prompts and constrained decoding/guardrails [85].
Deployment bias	Performance shifts from real-world use: workflow, user behavior, case-mix, site/sensor or prevalence changes.	Drift and misuse often go unseen; failures opaque and hard to attribute.	Track shifts in explanations (feature/importances by site/subgroup) to flag drift/misuse; still requires post-deployment monitoring, alerts, and periodic revalidation [81].
Interaction bias	Skewing of model due to interaction with it, e.g., through input of new data or rating/filtering made by users	Limited insight into black-box models raises the risk of such bias, particularly over time.	Specific design/tools used for explainability of importance, but generally much bigger chances of identifying and amending such bias [80].
Financial sustainability	Costs of training, evaluation, and maintenance of model (together with model-specific preprocessing)	Depending on model, preprocessing and training may be minimal or costly. Evaluation and maintenance costs are generally minimal, but potential debugging may inflict substantial difficulties and costs.	Depending on model, preprocessing and training may be minimal or costly. Evaluation may be costly. Maintenance costs are generally minimal.

XAI: explainable artificial intelligence, TCAV: Testing with Concept Activation Vectors, SHAP: SHapley Additive exPlanations

With that said, all XAI are not “created equally”. As previously mentioned, XAI may either be intrinsically explainable (and thereby interpretable; i.e., a white-box model) or explainable through “add-ons”, not seldom post-hoc. The latter category of XAI can never, by definition, be fully and completely accurate in explaining the model (otherwise it would simply be a white-box model) [24]. Rather, these techniques generally either provide summary level descriptions of the black-box model, or simplify the black-box model [4, 19]. These approximations may even lead to misinterpretation of the model’s actual inner workings. Thus, the level of fidelity (accuracy of explaining the model) needs to be taken into account. In some high-stake contexts, a fidelity even as high as 90% may be deemed insufficiently high. On the other hand, there may also be good reason for compromising explainability for the sake of performance and weigh the pros and cons at different levels/types of explainability. For example, it has been argued that it is ethically justifiable to use an ML model which is not (fully) explainable, but which consistently outperforms physicians, as long as it is possible to verify its soundness [24, 26]. Similarly, a study in the general public suggests that people prefer accuracy of AI over explainability [86]. The optimal balance is likely case-specific and dynamic over time and across cultural settings, and necessitates involvement of various stakeholders, including the public, methodologists/AI experts, policy makers, as well as healthcare providers and physicians.

## Personalized treatment of OSA based on treatable traits and XAI

In this section, we provide a general proposal of how XAI can be incorporated by physicians in the future for personalized treatment of patients with OSA. In general, we recommend that explainability should be favored higher relative to interpretability. The reason is simply that understanding of the specific infrastructure and underlying algorithmic patterns do not provide the same level of clinically relevant information as explaining how the model arrives at its output at a higher level of detail does. For a clinician, empirical validation and understanding of why certain outputs are given by AI for specific individual, and what factors likely influence/”drive” the model, is by far the most important perspective for usability, reflective in the overall practical goal of helping patients preserve/improve health and combat disease [87]. Interpretability, on the other hand, is of high relevance when it comes to debugging identified errors [24] and possibly for regulatory purposes (there is no consensus among experts regarding the actual requirement [and detail] of interpretability from the perspective of [data protection] law) [24]. Lacking mechanistic explanation is also nothing new to AI in the context of medical practice. Rather, history is filled with increasingly advanced technologies, including pharmaceuticals and diagnostic aids, for which physicians lack clear understanding of the inner workings but can nevertheless infer clinical utility through high-level validation data from trials, epidemiological studies, and professional experience [87].

**Fig. 3:**
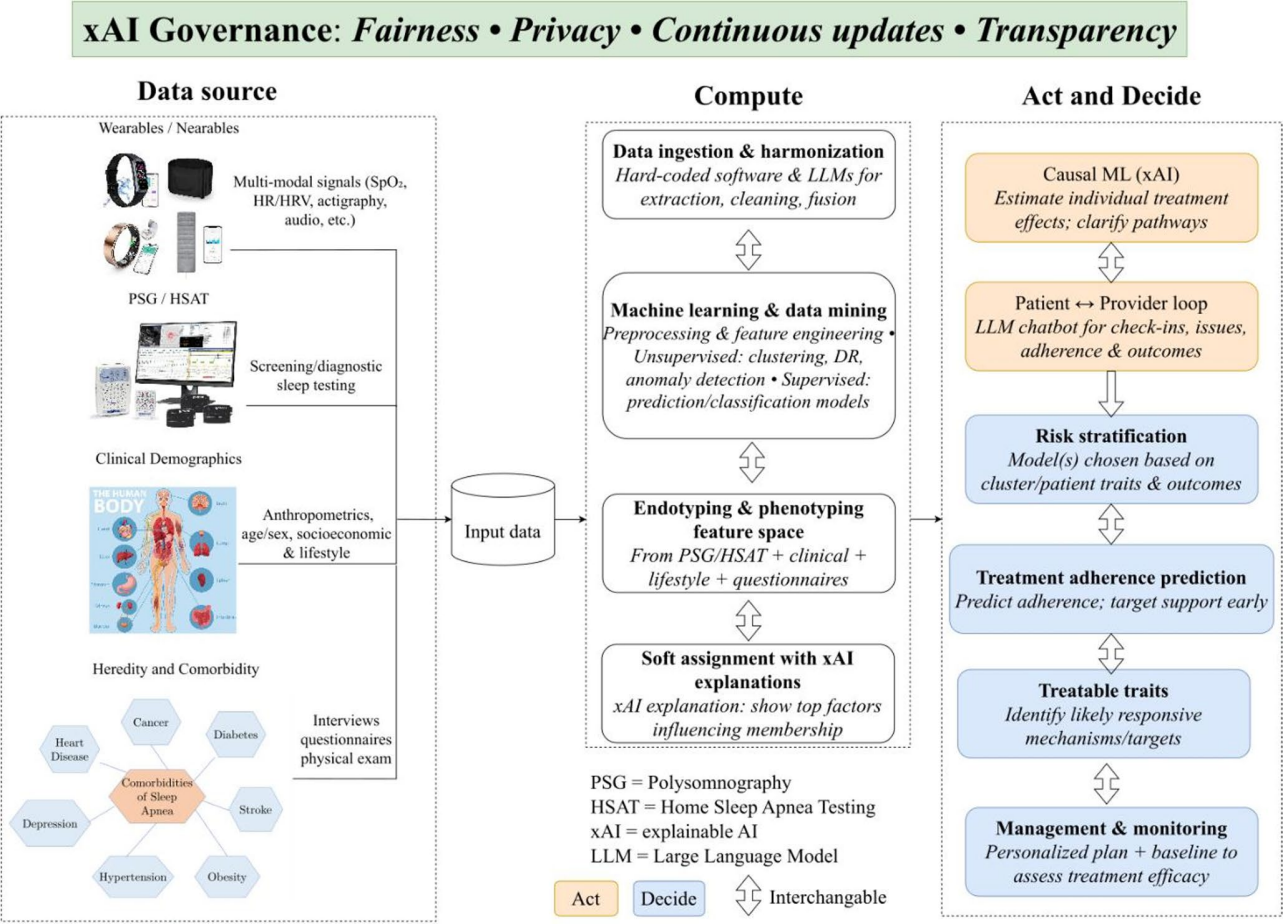
XAI-governed pipeline for OSA care: multimodal data (wearables/nearables, PSG/HSAT, demographics, comorbidities) are ingested and harmonized, feature-engineered, and softly clustered with explanations to drive causal-ML–informed risk stratification, adherence prediction, treatable-trait identification, and patient–provider loops—under fairness, privacy, continuous updates, and transparency

Figure 3 presents an XAI-governed approach in which AI distills the complex and heterogeneous, interconnected data that characterize sleep apnea—spanning etiologies, clinical presentations, comorbidity patterns, and treatment responses—into a data-driven, hypothesis-generating foundation for management. Within this framework, hardware plays a key role, as substantial multimodal data can be recorded through wearable/nearable devices, including readily available consumer sleep technologies. Following efficient screening and/or diagnostic testing, patients should undergo thorough endotyping and phenotyping based on conventional PSG or HSAT, as well as comprehensive supporting clinical data, including anthropometric, demographic, socioeconomic, and lifestyle (smoking, diet, alcohol, physical activity, etc.) variables; assessment of heredity of disease and present comorbidity (or risk factors/validated proxies) through interviews/questionnaires complemented by physical examination; and the patient’s subjective experience of health and sleep, along with goals and expectations for treatment [88]. These data can be collected with hard-coded software or LLMs and subsequently used as input to a clustering algorithm [89]. The output (cluster assignment) should preferably be probability-based, given the heterogeneity and overlapping features seen in OSA, and explainable so that the physician (together with the patient) can assess which factors most strongly influenced the degree of certainty for membership in each cluster. With sufficient model-training data, such information may also be used for risk stratification (with specific models used based on cluster/patient characteristics and outcomes of relevance), treatment-adherence prediction, identification of likely treatable traits, and—most importantly—serve as a baseline for assessing treatment efficacy on a data-driven, patient-centered foundation. Causal ML, where causal pathways between covariates are modeled and quantified explicitly, may be particularly suitable for clarifying individual treatment effects [90]. With XAI, physicians can help ensure that patients from underrepresented groups are modeled appropriately and that new information (e.g., novel diseases such as COVID-19) is incorporated as intended. XAI may also streamline connectivity between patients and clinicians—for example, via LLM chatbots through which patients can report issues as needed and receive/provide feedback on treatment effect and adherence [3]—which can then be distilled into concise, actionable recommendations for the treating physician. Given that in practice, OSA patients are managed by different specialists, many of which lack sufficient knowledge of the intricate multidimensional aspects of the disease, XAI offers a particularly impactful role, by unearthing e.g., comorbidity patterns that can be targeted as treatable traits with synergic effect [91], and also (perhaps most importantly) provide digestible justifications and rationale for recommendations, which can instill trust, aid in debugging abnormal recommendations or failed interventions, as well as increase sense of participation in patients. Z-syndrome, i.e., OSA with concomitant metabolic syndrome, is an illustrative example of patient subgroup for which personalized treatment with treatable traits using XAI can provide synergic benefits, as various clinical phenotypes may be seen and treatment options are manifold, including the use of pharmacologic agents, such as glucagone-like peptide-1 receptor agonists (GLP-1-RAs) [92]. Depending on patient factors and treatment goals, XAI applications can tailor management plans and be used for efficient follow-up by models being transparent and intuitive, and thus straightforward to cross-check with actual progress [93].

Given the probabilistic nature of LLMs and the limited insight into many complex black-box (and even XAI) algorithms, the performance of such algorithms (including the most advanced solutions available today, and likely so for the foreseeable future) will not be stable and precise enough to independently deal with the complex multidimensional contexts that constitute even the most typical OSA cases [94]. Rather, a physician will ultimately need to have the final say and judgement of the data at hand, although AI of various kinds may certainly streamline many processes, incorporate a richer yet digestible body of potentially actionable characterization data, and ultimately provide useful hypotheses and suggestions, through the role of a tireless around-the-clock assistant [95, 96].

### Limitations, research gaps, and future directions

One of the main risks with ML models is that they provide an output without declaring how sensible it is from a clinical perspective. For this reason, clustering and other ML-based modelling of clinical data requires assessment from physicians, as solely quantitative performance metrics may mask segmentations that are only sensible from a mathematical point of view. Similarly, most ML models are prone to overfitting, which hampers generalizability beyond the training data/population assessed. To combat these limitations, first, interdisciplinary co-operation needs to be improved and deepened in AI-based research within sleep medicine. Methodologists need to understand the needs, expectations, and evaluations of physicians, and vice versa. Second, efforts are needed to counteract the debilitating data privacy laws that overcomplicate even reasonable and safely performed pooling of clinical data. Here, federated learning (collaborative training of ML models without sharing raw data) [97]and differential privacy (mathematically provable secure obfuscation of data while maintaining overall patterns in the data) [98] may play an important enabling role. While XAI is increasingly recognized in sleep medicine research and recent years have seen increased publication rates of studies using XAI [99–101], the knowledge and utilization of such technologies remain scarce. Future research in the field of sleep apnea should also expand the horizon in terms of training data. One of the core strengths of AI is to analyze vast data, and this characteristic should be taken advantage of to a larger extent and be approached from a more purely data-driven perspective, where the data and AI analyses drive hypotheses and not the other way around. In practical terms, this means incorporating multi-modal data and more comprehensive sets of variables (although, of course, sensible preprocessing is always a must).

A diametral challenge to overfitting is the lacking representability of particularly large-scale and longitudinal clinical data. Most such data are collected in “Western” countries, where the populations are already in advantageous health positions. With the rapidly increasing development of consumer sleep technology, more affordable devices and public health strategies subsidizing distribution of such may expand much-needed large-scale data collection to rural and low-income populations [102]. Substantial proportions of the general population also maintain varying degrees of skepticism of AI [103]. Given the discrepancy between sociodemographic factors, such as education level (which in and of itself contributes to lower health literacy and increased risk of adverse health outcomes), and attitude towards AI [104], it is essential to increase the understanding (and thereby trust) of AI in the wider general population; XAI and efforts to standardize assessment methods of the fidelity of such technologies will play a key role in this matter.

To achieve these ambitious goals, timely and relevant education is a key stepping stone. Despite this, current curricula does not incorporate AI [105]. A recent ERS workshop report recognizes the need for AI to take on a bigger role in upcoming catalogues of knowledge and skills [106]. Indeed, the curricula of medical students and doctoral candidates must incorporate AI as well as XAI in particular. Focused workshops for professionals in sleep medicine are also warranted to provide up-to-date know-how and insights in how to combat common and novel challenges.

## Conclusions

AI is undoubtedly one of the most impactful technologies of our time, constituting a powerful tool to explore complex patterns in data throughout all stages of care-pathways of patients with SDB, from preventative, diagnostic, risk stratification to therapeutic and management optimization. XAI provides a further edge by enabling key stakeholders to gain insight into how the output of a model is obtained, however, not seldom at a cost. Physicians and future research endeavors should embrace XAI but also understand its limitation and maintain a pragmatic case-by-case balance of performance, clinical end-goals, patient perspectives, and ethical/legal perspectives.

## Data Availability

All data generated or analyzed during this study are included in the manuscript and its supplementary files. The manuscript has no associated data.
